# Comment on “Duration of rifampin therapy is a key determinant of improved outcomes in early-onset acute prosthetic joint infection due to Staphylococcus treated with a debridement, antibiotics and implant retention (DAIR): a retrospective multicenter study in France” by Becker et al. (2020)

**DOI:** 10.5194/jbji-6-17-2020

**Published:** 2020-07-22

**Authors:** Henk Scheper, Mark G. J. de Boer

**Affiliations:** Department of Infectious Diseases, Leiden University Medical Centre, Albinusdreef 2, 2333ZA, the Netherlands

Dear editor, The adjunctive role of rifampicin for staphylococcal prosthetic joint infection is an important and ongoing discussion. We compliment our colleagues for studying this important question in a multicenter collaboration (Becker et al., 2020). The authors conclude that prolonged duration of rifampicin therapy is a key determinant for improved outcomes in acute staphylococcal prosthetic joint infection treated with debridement, antibiotics and implant retention (DAIR). However, this conclusion seems to be flawed due to survival bias, exclusion bias and probably confounding by indication.

Survival bias is correctly mentioned by the authors. Rifampicin is often started 2 weeks after debridement when wounds are dry and antimicrobial sensitivity is known. All patients with early failures until start of rifampicin do not “survive” this period and will be assigned to the non-rifampicin group, leading to a skewed selection of failures in the non-rifampicin group. Correction for this bias is challenging and could be solved through optimal use of randomization methods. There are also other methods or designs.

Confounding by indication is inevitable in retrospective studies that aim to study treatment effects. For unclear reasons, 24 % of patients did not receive rifampicin, possibly because in this group drug–drug interactions or other comorbidities that may be independent risk factors for failure were present. Though the authors studied other factors associated with DAIR failure (smoking, diabetes mellitus, ASA score, rifampin combination therapy with a fluoroquinolone), residual confounding remains due to these factors for which correction is difficult (e.g., by propensity score methods under the condition that the correct variables were obtained).

The most important limitation of this study is that the authors decided to exclude DAIR failures occurring while the patient was still under rifampicin. Bias was not prevented but mistakenly introduced with this measure, as only failures in the rifampicin group can be excluded. This resulted in the observation of an even more skewed positive response in the group of patients receiving rifampicin.

**Figure 1:**
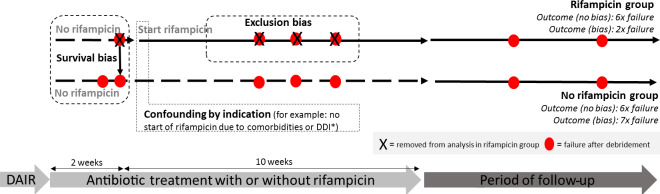
Hypothetical example of a prosthetic joint infection (PJI) study with a flawed outcome induced by methodological errors. Example of a PJI cohort, retrospectively stratified by use of rifampin. *Survival bias* occurs because only patients that “survive” the first weeks until the start with rifampin are analyzed in the rifampin group. All failures before start of rifampin will be analyzed in the non-rifampin group. *Confounding by indication *occurs when patients with certain risk factors for failure (e.g., comorbidities, drug–drug interactions, severely ill) are not selected for treatment with rifampin. *Exclusion bias* occurs if patients are excluded while they are still using rifampin, as only failures within the rifampin group can be excluded. In this hypothetical example assuming comparable treatment strategies, both groups would have an identical failure rate without bias (both six failures) but 3 times as much failure in the non-rifampin group after introduction of bias. ${}^{*}$ DDI: drug–drug interaction; f/u: follow-up. DAIR: debridement, antibiotics, implant retention.

Taken together, the results of this study should lead to a more cautious conclusion. Duration of rifampicin is associated with a better outcome, but this effect may be solely explained by introduction of bias by removing patients that failed on rifampicin treatment, confounding by indication and survival bias. Figure 1 shows how bias can potentially lead to erroneous conclusions in this type of observational cohort study. It is important to address these issues and to correct for them as much as possible upfront.

We completely agree with the authors that high-quality studies are warranted to elucidate the optimal duration of rifampicin as part of the antimicrobial therapy in patients with a staphylococcal PJI. A randomized controlled trial can answer the important question about the optimal duration of adjunctive use of rifampicin for a staphylococcal PJI.

## Data Availability

No data sets were used in this article.
